# The accumulation of multifunctional T cells in hypertrophied adenoids is dependent on age and related to clinical manifestation

**DOI:** 10.1186/2194-7791-1-S1-A18

**Published:** 2014-09-11

**Authors:** J Knolle, K Hebel, C Arens, C Hahne, G Jorch, M Brunner-Weinzierl

**Affiliations:** Department for Experimental Pediatrics und Neonatology, Otto-von-Guericke-University Magdeburg, Magdeburg, Germany; Department for Otorhinolaryngology, Otto-von-Guericke-University Magdeburg, Magdeburg, Germany

The quality of T cell responses differs dramatically during infancy and has been implicated in the susceptibility to infections, but the underlying cellular pathways are not well known. Here, we determined the effector phenotype of T cells in adenoids of 103 children with adenoid hypertrophy that underwent adenoidectomy. We compared children with sole upper airway obstruction with those suffering from concomitant otitis media with effusion or recurrent infections of the upper airways. Multifunctionality of T cells was determined as co-production of IFNγ, IL2, IL17 and TNFα using flow cytometry. Boolean gating was performed in FlowJo and analyzed using SPICE software. Statistical analysis was done by one-way-ANOVA.

We found that CD4 and CD8 T cells of all disease groups and at any examined age were able to co-produce all possible cytokine combinations. For CD8 T cells, patients exclusively showing upper airway obstruction accumulated 28% more triple and 30% more double producers compared with those suffering from subsequent infectious symptoms, while the latter had 15% more single producers. CD4 T cells showed similar results.

Analyzing the age-dependent changes in T-cell-quality, for CD4 T cells it can be stated that children of one and two years of age had 30% more double and 16% less single producers than older children, implying that multifunctionality increases with age. Additionally, for CD8 T cells children of at least three years had 47% more triple producers than two-year-olds. Children ≥3 years further had 38% more triple and 36% more double producers compared to younger children and 18% less single producers, suggesting a clear increase of cytokine co-expression between two and three years of age.

In conclusion, a correlation of multifunctional T cells with clinical symptoms can be established. Our data also show that two to three years of age are a critical period in an infant´s life to develop a higher quality of T cell responses.

